# 7-Diethyl­amino-2-propyl­sulfan­yl-3-(1*H*-1,2,4-triazol-1-yl)-4*H*-thio­chromen-4-one

**DOI:** 10.1107/S1600536812015954

**Published:** 2012-04-18

**Authors:** Chen Li, Guang Yan Yu, Xin Tian, Song Li, Tao Xiao

**Affiliations:** a Department of Applied Chemistry, College of Science, Nanjing University of Technology, Nanjing 210009, People’s Republic of China

## Abstract

In the title compound, C_18_H_22_N_4_OS_2_, the six-membered rings are almost coplanar, showing a dihedral angle between the mean planes of 9.0 (4)°, while the triazol ring is nearly perpendicular to the thio­chromen-4-one unit, making an angle of 89.8 (4)°. In the crystal, C—H⋯N hydrogen bonds link the mol­ecules in a stacked arrangement along the *c* axis.

## Related literature

For related compounds, see: Nohara *et al.* (1977 ▶); Xiao *et al.* (2010 ▶); Liu *et al.* (2011 ▶). For bond-length data, see: Allen *et al.* (1987 ▶).
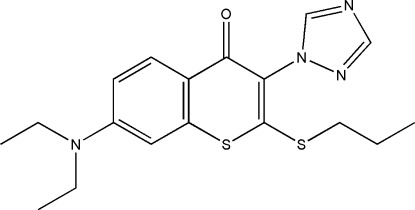


## Experimental

### 

#### Crystal data

C_18_H_22_N_4_OS_2_
*M* *_r_* = 374.52
Monoclinic, 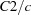

*a* = 21.937 (4) Å
*b* = 9.817 (2) Å
*c* = 19.059 (4) Å
β = 109.85 (3)°
*V* = 3860.6 (15) Å^3^
*Z* = 8
Mo *K*α radiation
μ = 0.29 mm^−1^
*T* = 293 K
0.30 × 0.20 × 0.20 mm

#### Data collection

Enraf–Nonius CAD-4 diffractometer
Absorption correction: ψ scan (North *et al.*, 1968 ▶) *T*_min_ = 0.918, *T*_max_ = 0.944
3644 measured reflections
3549 independent reflections
2309 reflections with *I* > 2σ(*I*)
*R*_int_ = 0.062
3 standard reflections every 200 reflections intensity decay: 1%

#### Refinement

*R*[*F* ^2^ > 2σ(*F* ^2^)] = 0.062
*wR*(*F* ^2^) = 0.181
*S* = 1.00
3549 reflections
226 parameters
1 restraint
H-atom parameters constrained
Δρ_max_ = 0.46 e Å^−3^
Δρ_min_ = −0.29 e Å^−3^

### 

Data collection: *CAD-4 Software* (Enraf–Nonius, 1985 ▶); cell refinement: *CAD-4 Software*; data reduction: *XCAD4* (Harms & Wocadlo, 1995 ▶); program(s) used to solve structure: *SHELXS97* (Sheldrick, 2008 ▶); program(s) used to refine structure: *SHELXL97* (Sheldrick, 2008 ▶); molecular graphics: *PLATON* (Spek, 2009) ▶; software used to prepare material for publication: *SHELXTL* (Sheldrick, 2008 ▶).

## Supplementary Material

Crystal structure: contains datablock(s) I, global. DOI: 10.1107/S1600536812015954/zq2162sup1.cif

Structure factors: contains datablock(s) I. DOI: 10.1107/S1600536812015954/zq2162Isup2.hkl

Supplementary material file. DOI: 10.1107/S1600536812015954/zq2162Isup3.cml

Additional supplementary materials:  crystallographic information; 3D view; checkCIF report

## Figures and Tables

**Table 1:** Hydrogen-bond geometry (Å, °)

*D*—H⋯*A*	*D*—H	H⋯*A*	*D*⋯*A*	*D*—H⋯*A*
C1—H1*A*⋯N4^i^	0.93	2.57	3.451 (5)	159

Symmetry code: (i) 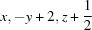.

**Fig. 1.**
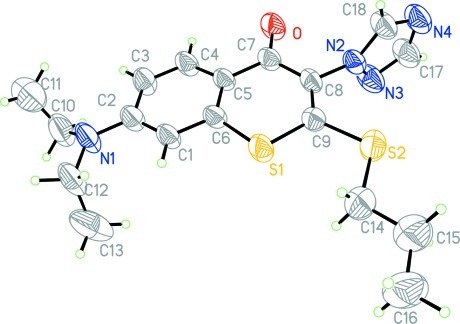
The molecular structure of the title compound. Displacement ellipsoids are drawn at the 50% probability level.

**Fig. 2.**
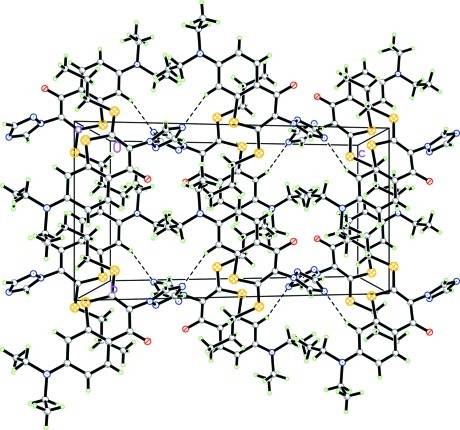
A packing diagram of the title compound. Hydrogen bonds are shown as dashed lines.

## supplementary crystallographic information

### Comment

The title compound is the key intermediate in the synthesis of a new kind of antiallergic drugs (Nohara *et al.*, 1977). The crystal structure determination has been carried out in order to elucidate its molecular conformation (Fig. 1).

The molecular structure of the title compound, C_18_H_22_O_1_N_4_S_2_, exhibits bond lengths and angles within normal ranges (Allen *et al.*, 1987; Xiao *et al.*, 2010; Liu *et al.*, 2011). The six-membered rings A (C1—C6) and B (S1/C6—C9) are almost coplanar, showing a dihedral angle between the mean planes of 9.0 (4)°. The dihedral angle between the ring A (C1—C6) and C (N2/N3/N4/C17/C18) is 83.0 (4)° while the dihedral angle between the ring B (S1/C6—C9) and C (N2/N3/N4/C17—C18) is 89.8 (4)°.

In the crystal structure, intermolecular C—H···N hydrogen bonds (Table 2) link the molecules in a stacked arrangement along the *c* axis (Fig. 2).

### Experimental

Diethylamine (13.8 ml, 134.4 mmol) was added to a solution of 2-(allylthio)-7-fluoro-3-(1*H*-1,2,4-triazol-1-yl)-4*H*-thiochromen-4-one (5 g, 15.7 mmol) in DMSO (30 ml) containing NaOH (1.8 g, 45 mmol). The yellow solution was stirred for about 12 h at room temperature. After completion of the reaction, the solution was poured into water (50 ml). The crystalline product was isolated by filtration and washed with water (300 ml). The precipitate was recrystallized with acetone and a yellow deposit was obtained (m.p. 420 K). Crystals suitable for X-ray analysis were obtained by dissolving the crude product (1.0 g) in ethanol (30 ml) and then allowing the solution to evaporate slowly at room temperature for about 7 d.

### Refinement

The H atoms were positioned geometrically with C—H = 0.93 Å for aromatic H atoms, with C—H = 0.97 Å for methylene H atoms, and with C—H = 0.96 Å for methyl H atoms. They were constrained to ride on their parent atoms with *U*_iso_(H) = 1.2*U*_eq_(C), or 1.5*U*_eq_(C) for the methyl H atoms.

### Crystal data

**Table:**

C_18_H_22_N_4_OS_2_	*F*(000) = 1584
*M**_r_* = 374.52	*D*_x_ = 1.289 Mg m^−^^3^
Monoclinic, *C*2/*c*	Melting point: 420 K
Hall symbol: -C 2yc	Mo *K*α radiation, λ = 0.71073 Å
*a* = 21.937 (4) Å	Cell parameters from 25 reflections
*b* = 9.817 (2) Å	θ = 9–13°
*c* = 19.059 (4) Å	µ = 0.29 mm^−^^1^
β = 109.85 (3)°	*T* = 293 K
*V* = 3860.6 (15) Å^3^	Block, yellow
*Z* = 8	0.30 × 0.20 × 0.20 mm

### Data collection

**Table:**

Enraf–Nonius CAD-4 diffractometer	2309 reflections with *I* > 2σ(*I*)
Radiation source: fine-focus sealed tube	*R*_int_ = 0.062
Graphite monochromator	θ_max_ = 25.4°, θ_min_ = 2.0°
ω/2θ scans	*h* = 0→26
Absorption correction: ψ scan (North *et al.*, 1968)	*k* = 0→11
*T*_min_ = 0.918, *T*_max_ = 0.944	*l* = −22→21
3644 measured reflections	3 standard reflections every 200 reflections
3549 independent reflections	intensity decay: 1%

### Refinement

**Table:**

Refinement on *F*^2^	Primary atom site location: structure-invariant direct methods
Least-squares matrix: full	Secondary atom site location: difference Fourier map
*R*[*F*^2^ > 2σ(*F*^2^)] = 0.062	Hydrogen site location: inferred from neighbouring sites
*wR*(*F*^2^) = 0.181	H-atom parameters constrained
*S* = 1.00	*w* = 1/[σ^2^(*F*_o_^2^) + (0.1*P*)^2^ + 2.5*P*] where *P* = (*F*_o_^2^ + 2*F*_c_^2^)/3
3549 reflections	(Δ/σ)_max_ < 0.001
226 parameters	Δρ_max_ = 0.46 e Å^−^^3^
1 restraint	Δρ_min_ = −0.29 e Å^−^^3^

### Special details

**Table:**

Geometry. All e.s.d.'s (except the e.s.d. in the dihedral angle between two l.s. planes) are estimated using the full covariance matrix. The cell e.s.d.'s are taken into account individually in the estimation of e.s.d.'s in distances, angles and torsion angles; correlations between e.s.d.'s in cell parameters are only used when they are defined by crystal symmetry. An approximate (isotropic) treatment of cell e.s.d.'s is used for estimating e.s.d.'s involving l.s. planes.
Refinement. Refinement of *F*^2^ against ALL reflections. The weighted *R*-factor *wR* and goodness of fit *S* are based on *F*^2^, conventional *R*-factors *R* are based on *F*, with *F* set to zero for negative *F*^2^. The threshold expression of *F*^2^ > σ(*F*^2^) is used only for calculating *R*-factors(gt) *etc*. and is not relevant to the choice of reflections for refinement. *R*-factors based on *F*^2^ are statistically about twice as large as those based on *F*, and *R*- factors based on ALL data will be even larger.

### Fractional atomic coordinates and isotropic or equivalent isotropic displacement parameters (Å^2^)

**Table:**

	*x*	*y*	*z*	*U*_iso_*/*U*_eq_	
O	0.31273 (13)	0.6959 (3)	−0.19491 (12)	0.0685 (7)	
S1	0.37800 (5)	0.93494 (9)	0.01442 (4)	0.0623 (3)	
C1	0.34042 (17)	0.7137 (3)	0.06581 (16)	0.0521 (8)	
H1A	0.3504	0.7653	0.1092	0.063*	
N1	0.31682 (18)	0.5173 (3)	0.12929 (16)	0.0768 (10)	
S2	0.42593 (6)	1.12341 (11)	−0.07537 (6)	0.0778 (4)	
N2	0.37390 (13)	0.9341 (3)	−0.19835 (14)	0.0517 (7)	
C2	0.31990 (18)	0.5786 (4)	0.06550 (18)	0.0575 (9)	
C3	0.3029 (2)	0.5069 (4)	−0.00294 (19)	0.0647 (10)	
H3A	0.2884	0.4175	−0.0051	0.078*	
N3	0.42763 (15)	0.9017 (4)	−0.21445 (17)	0.0755 (10)	
C4	0.30731 (19)	0.5662 (3)	−0.06570 (17)	0.0591 (9)	
H4A	0.2953	0.5160	−0.1097	0.071*	
N4	0.35895 (17)	1.0243 (4)	−0.30789 (17)	0.0752 (10)	
C5	0.32941 (15)	0.7002 (3)	−0.06632 (15)	0.0460 (7)	
C6	0.34602 (15)	0.7712 (3)	0.00137 (16)	0.0461 (7)	
C7	0.33381 (16)	0.7573 (3)	−0.13516 (16)	0.0498 (8)	
C8	0.36498 (16)	0.8890 (3)	−0.13097 (16)	0.0496 (8)	
C9	0.38666 (17)	0.9716 (3)	−0.07033 (17)	0.0521 (8)	
C10	0.3179 (3)	0.3638 (5)	0.1353 (3)	0.0961 (15)	
H10A	0.3371	0.3251	0.1009	0.115*	
H10B	0.3439	0.3366	0.1855	0.115*	
C11	0.2543 (3)	0.3150 (7)	0.1184 (3)	0.122 (2)	
H11A	0.2550	0.2174	0.1222	0.184*	
H11B	0.2288	0.3414	0.0685	0.184*	
H11C	0.2356	0.3527	0.1529	0.184*	
C12	0.3326 (2)	0.5939 (5)	0.1989 (2)	0.0825 (13)	
H12A	0.3115	0.5512	0.2304	0.099*	
H12B	0.3154	0.6856	0.1878	0.099*	
C13	0.4041 (3)	0.6019 (6)	0.2407 (3)	0.126 (2)	
H13A	0.4117	0.6538	0.2856	0.189*	
H13B	0.4253	0.6454	0.2101	0.189*	
H13C	0.4212	0.5117	0.2533	0.189*	
C14	0.4445 (2)	1.1997 (4)	0.0179 (2)	0.0828 (13)	
H14A	0.4048	1.2114	0.0289	0.099*	
H14B	0.4727	1.1394	0.0552	0.099*	
C15	0.4759 (3)	1.3310 (5)	0.0208 (3)	0.1027 (16)	
H15A	0.4476	1.3907	−0.0167	0.123*	
H15B	0.5155	1.3188	0.0095	0.123*	
C16	0.4921 (3)	1.3968 (5)	0.0972 (3)	0.1077 (17)	
H16A	0.5126	1.4832	0.0972	0.162*	
H16B	0.5209	1.3387	0.1343	0.162*	
H16C	0.4529	1.4102	0.1082	0.162*	
C17	0.4160 (2)	0.9580 (5)	−0.2799 (2)	0.0791 (13)	
H17A	0.4453	0.9527	−0.3056	0.095*	
C18	0.33441 (19)	1.0072 (4)	−0.25448 (18)	0.0632 (10)	
H18A	0.2946	1.0417	−0.2556	0.076*	

### Atomic displacement parameters (Å^2^)

**Table:**

	*U*^11^	*U*^22^	*U*^33^	*U*^12^	*U*^13^	*U*^23^
O	0.101 (2)	0.0720 (17)	0.0323 (11)	−0.0199 (14)	0.0227 (12)	−0.0092 (11)
S1	0.0961 (7)	0.0541 (5)	0.0374 (4)	−0.0189 (5)	0.0236 (4)	−0.0066 (4)
C1	0.075 (2)	0.0510 (19)	0.0301 (15)	−0.0042 (17)	0.0170 (15)	−0.0023 (14)
N1	0.126 (3)	0.0677 (18)	0.0389 (15)	−0.019 (2)	0.0313 (17)	0.0017 (14)
S2	0.1058 (9)	0.0682 (7)	0.0592 (6)	−0.0306 (6)	0.0277 (6)	−0.0010 (5)
N2	0.0590 (16)	0.0614 (17)	0.0362 (13)	−0.0001 (14)	0.0179 (12)	0.0061 (13)
C2	0.077 (2)	0.055 (2)	0.0401 (17)	−0.0026 (17)	0.0192 (16)	0.0015 (15)
C3	0.101 (3)	0.0449 (19)	0.0478 (19)	−0.0168 (19)	0.0249 (19)	−0.0029 (16)
N3	0.065 (2)	0.112 (3)	0.0568 (19)	0.0182 (19)	0.0302 (16)	0.0236 (18)
C4	0.089 (3)	0.0506 (19)	0.0336 (16)	−0.0120 (18)	0.0158 (16)	−0.0052 (15)
N4	0.082 (2)	0.096 (3)	0.0488 (17)	0.004 (2)	0.0244 (17)	0.0219 (17)
C5	0.0582 (19)	0.0483 (18)	0.0303 (14)	−0.0025 (15)	0.0133 (13)	−0.0011 (13)
C6	0.0564 (19)	0.0463 (17)	0.0336 (14)	0.0001 (14)	0.0129 (13)	−0.0008 (13)
C7	0.063 (2)	0.0542 (19)	0.0323 (15)	−0.0034 (16)	0.0159 (14)	−0.0033 (14)
C8	0.060 (2)	0.0570 (19)	0.0323 (15)	−0.0012 (16)	0.0167 (14)	0.0056 (14)
C9	0.064 (2)	0.0488 (19)	0.0397 (17)	−0.0095 (16)	0.0117 (15)	0.0002 (14)
C10	0.146 (5)	0.086 (2)	0.060 (3)	−0.022 (3)	0.040 (3)	0.009 (2)
C11	0.140 (5)	0.142 (5)	0.081 (4)	−0.004 (4)	0.033 (3)	0.006 (3)
C12	0.132 (4)	0.078 (3)	0.043 (2)	−0.009 (3)	0.037 (2)	0.0025 (19)
C13	0.162 (6)	0.125 (5)	0.059 (3)	0.014 (4)	−0.003 (3)	0.006 (3)
C14	0.107 (3)	0.068 (3)	0.069 (3)	−0.022 (2)	0.024 (2)	−0.003 (2)
C15	0.116 (4)	0.083 (3)	0.094 (4)	−0.010 (3)	0.016 (3)	−0.003 (3)
C16	0.135 (5)	0.082 (3)	0.089 (4)	−0.010 (3)	0.016 (3)	−0.024 (3)
C17	0.076 (3)	0.117 (4)	0.053 (2)	0.008 (3)	0.033 (2)	0.023 (2)
C18	0.069 (2)	0.077 (2)	0.0439 (18)	0.007 (2)	0.0201 (17)	0.0133 (18)

### Geometric parameters (Å, º)

**Table:**

O—C7	1.231 (4)	C8—C9	1.358 (4)
S1—C9	1.727 (3)	C10—C11	1.405 (7)
S1—C6	1.738 (3)	C10—H10A	0.9700
C1—C6	1.394 (4)	C10—H10B	0.9700
C1—C2	1.400 (5)	C11—H11A	0.9600
C1—H1A	0.9300	C11—H11B	0.9600
N1—C2	1.378 (4)	C11—H11C	0.9600
N1—C12	1.460 (5)	C12—C13	1.502 (7)
N1—C10	1.512 (6)	C12—H12A	0.9700
S2—C9	1.740 (3)	C12—H12B	0.9700
S2—C14	1.843 (4)	C13—H13A	0.9600
N2—C18	1.335 (4)	C13—H13B	0.9600
N2—N3	1.353 (4)	C13—H13C	0.9600
N2—C8	1.433 (4)	C14—C15	1.454 (6)
C2—C3	1.416 (5)	C14—H14A	0.9700
C3—C4	1.363 (4)	C14—H14B	0.9700
C3—H3A	0.9300	C15—C16	1.520 (6)
N3—C17	1.308 (4)	C15—H15A	0.9700
C4—C5	1.404 (4)	C15—H15B	0.9700
C4—H4A	0.9300	C16—H16A	0.9600
N4—C18	1.314 (4)	C16—H16B	0.9600
N4—C17	1.350 (5)	C16—H16C	0.9600
C5—C6	1.401 (4)	C17—H17A	0.9300
C5—C7	1.460 (4)	C18—H18A	0.9300
C7—C8	1.452 (5)		
			
C9—S1—C6	103.07 (15)	C10—C11—H11A	109.5
C6—C1—C2	120.3 (3)	C10—C11—H11B	109.5
C6—C1—H1A	119.9	H11A—C11—H11B	109.5
C2—C1—H1A	119.9	C10—C11—H11C	109.5
C2—N1—C12	120.6 (3)	H11A—C11—H11C	109.5
C2—N1—C10	119.8 (3)	H11B—C11—H11C	109.5
C12—N1—C10	116.9 (3)	N1—C12—C13	113.0 (4)
C9—S2—C14	104.25 (17)	N1—C12—H12A	109.0
C18—N2—N3	108.8 (3)	C13—C12—H12A	109.0
C18—N2—C8	129.4 (3)	N1—C12—H12B	109.0
N3—N2—C8	121.7 (3)	C13—C12—H12B	109.0
N1—C2—C1	121.4 (3)	H12A—C12—H12B	107.8
N1—C2—C3	121.3 (3)	C12—C13—H13A	109.5
C1—C2—C3	117.3 (3)	C12—C13—H13B	109.5
C4—C3—C2	121.4 (3)	H13A—C13—H13B	109.5
C4—C3—H3A	119.3	C12—C13—H13C	109.5
C2—C3—H3A	119.3	H13A—C13—H13C	109.5
C17—N3—N2	102.4 (3)	H13B—C13—H13C	109.5
C3—C4—C5	122.4 (3)	C15—C14—S2	110.1 (3)
C3—C4—H4A	118.8	C15—C14—H14A	109.6
C5—C4—H4A	118.8	S2—C14—H14A	109.6
C18—N4—C17	101.6 (3)	C15—C14—H14B	109.6
C6—C5—C4	116.2 (3)	S2—C14—H14B	109.6
C6—C5—C7	124.1 (3)	H14A—C14—H14B	108.2
C4—C5—C7	119.7 (3)	C14—C15—C16	111.5 (4)
C1—C6—C5	122.4 (3)	C14—C15—H15A	109.3
C1—C6—S1	113.6 (2)	C16—C15—H15A	109.3
C5—C6—S1	123.9 (2)	C14—C15—H15B	109.3
O—C7—C8	120.6 (3)	C16—C15—H15B	109.3
O—C7—C5	121.6 (3)	H15A—C15—H15B	108.0
C8—C7—C5	117.8 (3)	C15—C16—H16A	109.5
C9—C8—N2	117.6 (3)	C15—C16—H16B	109.5
C9—C8—C7	126.9 (3)	H16A—C16—H16B	109.5
N2—C8—C7	115.5 (3)	C15—C16—H16C	109.5
C8—C9—S1	123.6 (3)	H16A—C16—H16C	109.5
C8—C9—S2	120.1 (3)	H16B—C16—H16C	109.5
S1—C9—S2	116.33 (18)	N3—C17—N4	115.7 (3)
C11—C10—N1	109.5 (5)	N3—C17—H17A	122.1
C11—C10—H10A	109.8	N4—C17—H17A	122.1
N1—C10—H10A	109.8	N4—C18—N2	111.4 (3)
C11—C10—H10B	109.8	N4—C18—H18A	124.3
N1—C10—H10B	109.8	N2—C18—H18A	124.3
H10A—C10—H10B	108.2		
			
C12—N1—C2—C1	2.4 (6)	N3—N2—C8—C9	90.7 (4)
C10—N1—C2—C1	−158.5 (4)	C18—N2—C8—C7	89.7 (4)
C12—N1—C2—C3	−178.1 (4)	N3—N2—C8—C7	−88.7 (4)
C10—N1—C2—C3	21.0 (6)	O—C7—C8—C9	175.5 (3)
C6—C1—C2—N1	177.0 (3)	C5—C7—C8—C9	−5.4 (5)
C6—C1—C2—C3	−2.5 (5)	O—C7—C8—N2	−5.1 (5)
N1—C2—C3—C4	−178.5 (4)	C5—C7—C8—N2	173.9 (3)
C1—C2—C3—C4	1.0 (6)	N2—C8—C9—S1	178.3 (2)
C18—N2—N3—C17	−0.3 (4)	C7—C8—C9—S1	−2.3 (5)
C8—N2—N3—C17	178.4 (3)	N2—C8—C9—S2	−2.0 (4)
C2—C3—C4—C5	0.7 (6)	C7—C8—C9—S2	177.4 (3)
C3—C4—C5—C6	−0.8 (5)	C6—S1—C9—C8	6.2 (4)
C3—C4—C5—C7	179.2 (4)	C6—S1—C9—S2	−173.5 (2)
C2—C1—C6—C5	2.5 (5)	C14—S2—C9—C8	179.0 (3)
C2—C1—C6—S1	−175.3 (3)	C14—S2—C9—S1	−1.2 (3)
C4—C5—C6—C1	−0.7 (5)	C2—N1—C10—C11	−98.6 (5)
C7—C5—C6—C1	179.2 (3)	C12—N1—C10—C11	99.8 (5)
C4—C5—C6—S1	176.8 (3)	C2—N1—C12—C13	−82.1 (5)
C7—C5—C6—S1	−3.2 (5)	C10—N1—C12—C13	79.4 (5)
C9—S1—C6—C1	174.2 (3)	C9—S2—C14—C15	−178.2 (4)
C9—S1—C6—C5	−3.6 (3)	S2—C14—C15—C16	−179.9 (4)
C6—C5—C7—O	−172.8 (3)	N2—N3—C17—N4	−0.1 (5)
C4—C5—C7—O	7.2 (5)	C18—N4—C17—N3	0.5 (5)
C6—C5—C7—C8	8.2 (5)	C17—N4—C18—N2	−0.6 (5)
C4—C5—C7—C8	−171.9 (3)	N3—N2—C18—N4	0.6 (5)
C18—N2—C8—C9	−90.9 (4)	C8—N2—C18—N4	−177.9 (3)

### Hydrogen-bond geometry (Å, º)

**Table:**

*D*—H···*A*	*D*—H	H···*A*	*D*···*A*	*D*—H···*A*
C1—H1*A*···N4^i^	0.93	2.57	3.451 (5)	159

Symmetry code: (i) *x*, −*y*+2, *z*+1/2.
